# Micro/Nano Electrode Array Sensors: Advances in Fabrication and Emerging Applications in Bioanalysis

**DOI:** 10.3389/fchem.2020.573865

**Published:** 2020-11-13

**Authors:** Yang Liu, Xiuting Li, Jie Chen, Chonglin Yuan

**Affiliations:** ^1^Institute for Advanced Study, Shenzhen University, Shenzhen, China; ^2^College of Physics and Optoelectronic Engineering, Shenzhen University, Shenzhen, China

**Keywords:** micro/nano electrode array, electrochemical sensor, array sensors fabrication, biological application, smart sensing

## Abstract

Due to the rapid development of micro/nano manufacturing techniques and the greater understanding in electrochemical principles and methods, micro/nano electrode array sensing has received much attention in recent years, especially in bioanalysis. This review aims to explore recent progress in innovative techniques for the construction of micro/nano electrode array sensor and the unique applications of various types of micro/nano electrode array sensors in biochemical analysis. Moreover, the new area of smart sensing benefited from miniaturization of portable micro/nano electrode array sensors as well as wearable intelligent devices are further discussed.

## Introduction

Electrochemical arrays, containing numbers of sensors on single platform or device, are of great interest in electroanalytical chemistry since quantification and characterization of substances in complex sample can be conducted simultaneously with the individual sensors based on electrochemical analysis at high time resolution and sensitivity (LaFratta and Walt, 2008; Chow et al., 2009; Park et al., 2011; Takulapalli et al., 2012; Fu et al., 2016; Li et al., 2019). Electrochemical sensing has a long history since the first electrochemical sensors for oxygen were reported in 1960's and later glucose sensors developed in 2002 (Wang, 2002). Benefited from the great progress made in micro and nano fabrication technology, the field of electrochemical sensing is experiencing a revival (Lemay and White, 2016). Especially in last few years, owing to the rapid development in micro/nano meter scale machining technology, micro/nano electrode array sensors emerged and are constantly receiving great attention because of their multiplexing ability and robustness for bioanalysis at different biological levels (e.g., cell, tissue or organ, etc.) as well as *in-situ* and real-time dynamic monitoring with higher spatiotemporal resolution and selectivity (Arrigan, 2004; Godino et al., 2009; Ongaro and Ugo, 2013; Liu et al., 2017; Du et al., 2019). Moreover, due to the breakthrough on new materials and microelectronic technology in recent years, micro/mano electrode array sensors are moving toward miniaturization, digitization, intelligence and systematization, and are widely used in diverse fields including environmental monitoring, medical and health care (Feeney and Kounaves, 2000; Berduque et al., 2007; Orozco et al., 2010; Sekretaryova et al., 2015; Liu et al., 2017).

**Figure 1:**
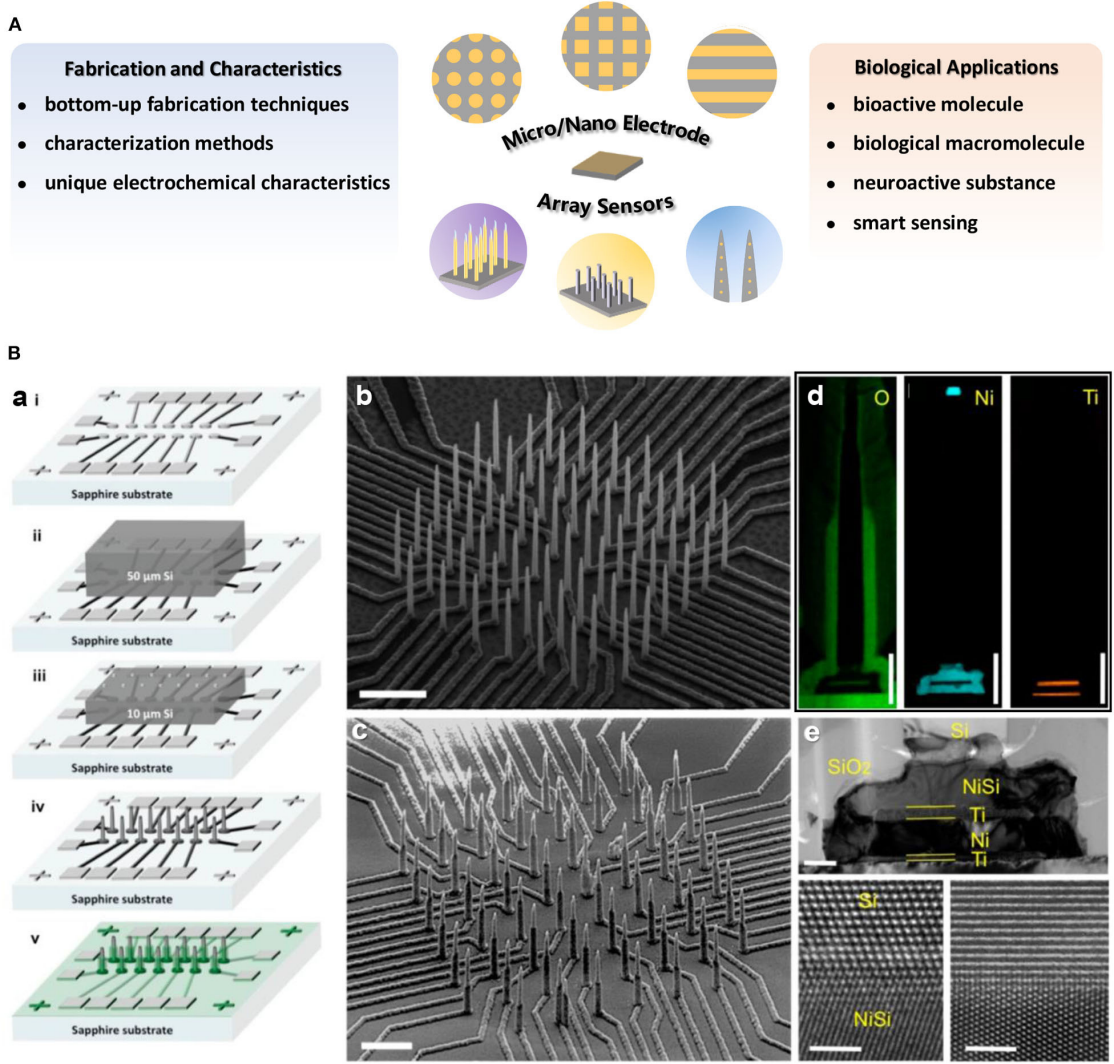
**(A)** Overview of the main research content on micro/nano electrode array sensors introduced in this minireview. **(B) (a)** Illustration of fabrication procedure for high density electrically isolated nanowire probes. The SEM images of a typical Si nanowire array **(b)** after etching and **(c)** after SiO_2_ passivation. Scale bar in **(b)** is 5 μm and in **(c)** is 3 μm. **(d)** EDX mapping of O, Ni, and Ti on single nanowire. Scale bars are 1 μm. **(e)** A TEM image of the NiSi/Ti/Ni/Ti underneath the Si nanowire. Scale bar is 200 nm. The bottom panels are HRTEM images at the interface between Si and NiSi. Scale bars in bottom panels are 2 nm. Liu et al. (2017). Copyright 2017 American Chemical Society.

Several reviews have summarized the progresses on micro- or nano-electrode array sensors with concerns for relevant theory, fabrication or application (Arrigan, 2004; Huang et al., 2009; Orozco et al., 2010; Yeh and Shi, 2010; Zoski and Wijesinghe, 2010; Henstridge and Compton, 2012; Chen et al., 2013; Ongaro and Ugo, 2013; Tomčík, 2013; Karimian et al., 2016; Karimian and Ugo, 2019). In this minireview (Figure 1A), we have mainly focused on the recent accomplishments in materials and innovative techniques for the construction of various micro/nano electrode array sensors and their unique applications in bioanalysis. In addition, the recent development of smart sensing and wearable intelligent devices benefited from miniaturization of portable micro/nano electrode array sensors are further discussed.

## Fabrication and Characteristics of Micro/Nano Electrode Arrays

Single micro/nano electrodes, with dimensions at micro/nano scale, show unique advantages, such as increased mass transport, faster transient response as well as lower destructive probe, compared to conventional electrodes (Penner et al., 1990; Amatore, 1995). A certain number of micro/nano electrodes are arranged and combined to form micro/nano electrode arrays. The electrode size, morphology, structure and material generally determine the electrochemical performance of micro/nano electrode arrays (Arrigan, 2004; Chevallier and Compton, 2007; Hood et al., 2009). Various types of micro/nano electrode arrays adapted to different measuring conditions have been designed and fabricated to meet the sensing requirement. In general, micro/nano electrode arrays could be prepared by means of bottom-up fabrication techniques, mainly involving electrode material (metal, carbon, ceramic, etc.) layer deposition or growth on the top, bottom, or formation in-between sandwich structure relative to the templates or substrates (silicon, glass, polymer, ceramic, etc.) (Patel et al., 2008; Xiang et al., 2009; Lee and Silvester, 2016; Ledo et al., 2017). Photolithography is basic technology commonly applied for micro electrode arrays manufacturing, including surface insulation and micron holes drilling based on parts of thin film or the bulk of a substrate removal selectively by photoresists and illumination sources exposure (Lowinsohn et al., 2006; Aguiar et al., 2007; Ordeig et al., 2008; Xu et al., 2008). Other preparation processes such as screen print, deposition, membrane formation, firing, etc. have also been used for micro electrode arrays fabrication (Mann and Mikkelsen, 2008; Vagin et al., 2014; Lee and Silvester, 2016). In addition, ink-jet and 3D printing technologies have recently become a powerful alternative processing tool for high-resolution microstructures which enables complex electrode patterns at micro scale (Nouran et al., 2018; Kundu et al., 2019). Based on the above various fabrication methods, different types of micro electrode arrays (e.g., microdisk or microband electrode array and interdigitated, linear or 3D micro electrode array, etc.) have been reported (Fiaccabrino et al., 1996; Aguiar et al., 2007; Ordeig et al., 2008; Xu et al., 2008; Menshykau et al., 2010; Yi et al., 2016).

With the breakthrough of nano processing technology and electrochemical instrumentation with higher performance, nano electrode arrays have aroused wider research interests. So far, beside photolithography (Xiang et al., 2009; Chen et al., 2011; Heo et al., 2011), other methods for nano electrode arrays manufacturing, include nanoimprint-lift-off, focused ion beam (FIB), electron beam lithography (EBL) (Sandison and Cooper, 2006; Lanyon and Arrigan, 2007; Errachid et al., 2008; Moretto et al., 2011; Branagan et al., 2012; Ma et al., 2013; Wahl et al., 2013; Sentic et al., 2016). For example, via FIB milling following by a layer-by-layer deposition, nano electrode arrays on nanochannels with embedded annular nanoband electrodes have been prepared (Branagan et al., 2012).

Nano electrode arrays can be constructed on solid substrates by chemical means, such as template-based method involving electrochemical deposition or chemical plating process (Zhang et al., 2004; Cao and Liu, 2008; Ongaro et al., 2012). In addition, nucleation and growth of materials to form nanostructures with electrochemical performances has also become a way for construction of nano electrode arrays. Vertically aligned carbon nanotubes/nanofibers (Arumugam et al., 2009; Robinson et al., 2016; Song et al., 2019) as well as a vast range of nano electrocatalysts (e.g., prussian blue, porous gold or platinum nanowire, NiO nanocone, mesoporous rhombus-shaped ZnO rod, etc.) have been fabricated as nano electrode arrays (Karyakin et al., 2004; Puganova and Karyakin, 2005; Zhang et al., 2009; Wang et al., 2011, 2012; Wen et al., 2015). Different fabrication techniques can be combined to construct more complex nano electrode arrays. For instance, by integrating track-etched polycarbonate membrane and a lithographically fabricated addressable Pt ultramicroelectrode array platform, microregions of a macro-nanoelectrode membrane could be individually addressed (Zoski et al., 2007). Furthermore, an integration scheme for high-density individually and electrically addressable out-of-plane Si nanowire arrays by solid-state wafer bonding were developed for the first time. The fabrication procedures of these new type nano electrode arrays with submicrometer site-to-site spacing mainly involved a combination of photolithography, EBL and plasma enhanced chemical vapor deposition (PECVD) atop an electrically insulating and transparent sapphire substrate with standard integrated circuit fabrication technologies (Liu et al., 2017).

Variety of methods have been developed and employed to achieve electrode arrays characterization at the micron or nano scale, which is helpful for better understanding of electrochemical performance of electrode arrays. Scanning electron microscopy or transmission electron microscopy (SEM, TEM) are commonly used to visualize the dimensions or morphologies of micro/nano electrode arrays (Figure 1B). The topography of the nano electrode arrays is observed by atomic force microscope (AFM) (Puganova and Karyakin, 2005), and *in situ* AFM technique is even applied to characterize the geometry and surface reactivity variation of electrodes during working in solution (Nogala et al., 2012). X-ray photoelectron spectroscopy (XPS) (Forrer et al., 2000) or energy-dispersive X-ray spectroscopy (EDX) (Liu et al., 2017) normally be used for arrays surface element composition identification (Figure 1B). Additionally, the steady-state limiting current can reflect electrode morphology to some extent (Bond et al., 1988; Arrigan, 2004; Wahl et al., 2013), and the diffusion or reaction layers at nano electrodes arrays can be studied from electrochemical luminescence imaging (Sentic et al., 2016).

Under micro/nano scale sizes, electrode arrays show unique electrochemical characteristics compared with conventional electrodes. As the electrode dimensions decrease to micro/nano scale, the double layer has lower capacitance, and smaller time constant enable micro/nano electrode arrays to achieve rapid response and high-speed measurement under less destructive sensing (Freeman et al., 2013). Electrode radius becomes smaller than the thickness of diffusion layer for micro/nano electrode arrays, mass transport increases, which is appropriate for the study of electrochemical process transients (Godino et al., 2009; Henstridge and Compton, 2012). For the nano electrode arrays obtained by EBL, important advantages, such as exactly controlled geometry and the miniaturization possibility from pure radial diffusion regime, are showed (Moretto et al., 2011). The total current generated from micro/nano electrode arrays is the sum of each micro/nano electrodes, resulting in increased detection current, improved signal-to-noise environment and higher analysis sensitivity (Bond et al., 1988; Arrigan, 2004). Furthermore, by electrode modification with specific recognition sites, functional molecules or materials, the stability and selectivity of micro/nano electrode arrays can be improved (Fruk et al., 2007; Arya et al., 2010; Frey et al., 2010; Pang et al., 2017). For instance, as a layered metal oxide semiconductor equipped with high work function and good hole conductivity, MoO_3_ has been electrodeposited successfully on the surfaces of as-prepared TiO_2_ nanoneedles (NNs) to constitute TiO_2_ NNs@MoO_3_ array. The latter shows ultrasensitive photocurrent response and a wide linear range with a low detection limit on account of tuned interfacial microstructure (Pang et al., 2017). Note that due to the significant increase in mass transfer rate when the size of electrode reaches nano scale, the resulted extreme sensitivity to the electron transfer kinetics may limit their performance in biosensing applications (Menon and Martin, 1995; Sun and Mirkin, 2006; Sliusarenko et al., 2015, 2017; Yu et al., 2016; Edwards et al., 2018).

## Micro/Nano Electrode Array Sensors for Biological Application

**Figure 2:**
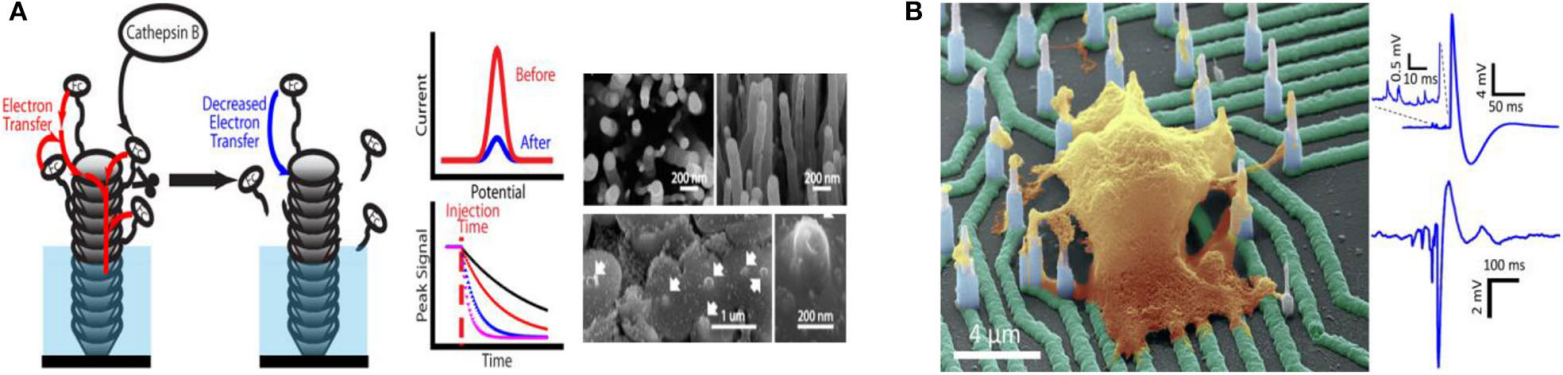
**(A)** Carbon nanofiber nanoelectrode arrays and their electrochemical activity for protease analysis (Song et al., 2019). Copyright 2019 American Chemical Society. **(B)** Recording from hiPSC-derived cortical neurons on high density individually addressable nanowire arrays. Liu et al. (2017). Copyright 2017 American Chemical Society.

With the rapid development of science and technology, humans are increasingly focusing on individual health and the impact of environmental conditions on life entities and activities. Over recent decades, studies of bioactive related chemicals (e.g., inorganic salt, neuroactive substances, carbohydrate, nucleic acid, proteins, gas, etc.) have attracted widespread interest due to their crucial roles in a series of physiological and pathological processes, as well as biological applications. Distinguishing characteristics enable micro/nano electrode arrays to act as effective electrochemical sensors for biological application, promoting *in vitro* and *in vivo* biosensing research. Various typical applications with micro/nano electrode arrays in bioanalysis are showed in Table 1. Micro/Nano electrode arrays have been applied to electrochemical sensing for bioactive molecule with high sensitivity and selectivity (Puganova and Karyakin, 2005; Burmeister et al., 2008; Xu et al., 2008; Jiang and Zhang, 2009; Arya et al., 2010; Frey et al., 2010; Gholizadeh et al., 2012; Wang et al., 2012; Hinzman et al., 2015; Zhang et al., 2016). Gholizadeh et al. (2012) employed a high-density vertically aligned carbon nanotube nano electrode array (VACNT-NEA) with glutamate dehydrogenase covalently attached on the CNT tips as electrochemical glutamate biosensors, exhibiting an extremely low detection limit of 10 nM for glutamate. A ceramic-based multisite micro electrode array was developed by Burmeister et al. (2008) for simultaneous determinations of choline and acetylcholine in the central nervous system. The array was designed with one recording site modified with choline oxidase (ChOx) and the other with acetylcholinesterase and ChOx. Hinzman et al. (2015) reported the selectively measurement of extracellular adenosine by using an enzyme-linked microelectrode array A *in vivo* limit of detection leveled down to ~0.04 μM is achieved. Moreover, biological macromolecules (e.g., DNA, RNA, protease, etc.) can be detected by micro/nano electrode array sensors (Koehne et al., 2004; Lapierre-Devlin et al., 2005; Periyakaruppan et al., 2013; Silvestrini et al., 2013; Selvam et al., 2015; Lee et al., 2016; Delle et al., 2018; Song et al., 2019). Song et al. (2019) developed vertically aligned carbon nanofibers as unique electrochemical platform for investigating protease activities (Figure 2A). The carbon nanofibers are functionalized with specific peptide substrates containing a ferrocene tag. It is reported that the detection limit for cathepsin B activity and concentration are 2.49 × 10^−4^ s^−1^ and 0.32 nM, respectively. The fabricated nano electrode arrays showed outstanding selectivity with negligible cross-reaction with 6.0 nM of other two cancer-related proteases (ADAM10 and ADAM17). It demonstrates that the electrochemical chip fabricated with present methodology holds great potential in rapid profiling protease activities in cancer diagnosis. Micro/Nano electrode arrays have been employed as immunosensors, including sensitive detection for immunoglobulin IgY (Bottari et al., 2014), cardiac biomarker (Sharma et al., 2018) and IgG-type tissue transglutaminase (Habtamu et al., 2019), which show the potential to be applied for the diagnosis and monitor of diseases in clinical or nonclinical settings.

**Table 1:** Micro/Nano electrode arrays for bioanalysis applications.

**Electrode array type**	**Fabrication**	**Analysis target**	**Measurement methods**	**Sensing performance**	**References**
VACNT-NEA	Photolithography/glutamate dehydrogenase immobilization	Glutamate	Differential pulse voltammograms (DPV)	10 nM (LOD)	Gholizadeh et al., 2012
Enzyme-linked and self-referenced microelectrode arrays	Four Pt recording sites linked with ADA enzyme/ micropipette attachment	Extracellular adenosine	Constant potential amperometry	0.04 μM (*in vivo* LOD)	Hinzman et al., 2015
Au-coated vertical silicon nanowire electrode array (VSNEA)	Chemical vapor deposition (CVD)/ peptide immobilization and RNA functionalization	HIV-1 RRE RNA	DPV	1.513 fM (LOD)	Lee et al., 2016
Gold nanoscale interdigitated electrode (IDE) arrays	Nanoimprint and photolithography	DNA hybridization	Impedance spectroscopy	Dynamic detection range of 1–100 nM	Delle et al., 2018
Vertically aligned carbon nanofibers (VACNFs) arrays	PECVD/passivation and functionalization of Fc-hexapeptide substrates	Cathepsin B activity and concentration	AC voltammetry (ACV)	2.49 × 10^−4^ s^−1^ and 0.32 nM (LOD)	Song et al., 2019
Substrate-bound interdigitated array (IDA) nanoelectrodes.	Photolithography and RF-sputtering/ immobilization of mAb-cMyo	Cardiac myoglobin (cMyo).	Cyclic voltammetry (CV)	Linear detection range of 0.001–100 ng/mL 0.43 pg/mL (LOD)	Sharma et al., 2018
Cylindrical gold nano electrode arrays (CAuNE)	Laser interference lithography (LIL) and electrochemical deposition (ECD)	Dopamine (DA) in human neural cells	CV	5.83 μM (LOD)	Kim et al., 2018
Four-shank implantable micro-electrode array	Photolithography/ Pt/rGO nanocomposites modification onto the recording microelectrode sites	Synchronous DA levels and neural spike real-time detection	Amperometry	<20 nM (LOD)	Xiao et al., 2019

Micro/Nano electrode array sensing technology provides an effective tool for neuroactive substances detection and neuron activities direct reading, contributing to reveal the complex neuron communication and connection (Burmeister et al., 2008; Dincer et al., 2015; Liu et al., 2017; Kim et al., 2018; Ledo et al., 2018; Du et al., 2019; Xiao et al., 2019). Kim et al. (2018) developed a simple cylindrical gold nano electrode arrays with optimized electrode size and height for measuring dopamine and detecting its release from human dopaminergic neurons. By modulating the dopamine (DA) concentration, Parkinson's disease (PD) can be well-treated. In order to improve electron transmission capabilities, Xiao et al. (2019) designed a four-shank implantable micro-electrode array with platinum nanoparticles and reduced graphene oxide nanocomposites (Pt/rGO) fabricated onto the recording microelectrode sites. Synchronous DA levels and neural spike real-time detection was achieved in the cortex and caudate putamen during apomorphine modulation of 6-hydroxydopamine-induced Parkinson's disease rats. The changes of ion channel currents and intracellular potentials originated from the ion concentrations differences (Na^+^ and K^+^) between the inside and outside of the cell reveal the response of neurons to drugs. Liu et al. (2017) utilized a novel high-density vertical Si nanowire arrays with independent electrical addressability and superior spatial resolution to conduct electrophysiological recordings from mouse and rat primary neurons, as well as human induced pluripotent stem cell-derived neurons (Figure 2B). High signal-to-noise ratios and sensitivity to potentials (as low as a few millivolts) without cell damage was achieved. This new nano electrode arrays is expected to be a platform for drugs screening based on the disease models of neuronal networks, helping to better understand the communication of individual cells in large areas of neural networks and the mechanisms of the drug treatment to neurological diseases.

Owing to the breakthrough in new materials, microelectronic technology as well as electrochemical understandings, biosensors based on micro/nano electrode arrays are moving toward miniaturization, digitization, intelligence and systematization. Smart sensing created from miniaturization of portable micro/nano electrode array sensors as well as wearable intelligent devices has been paid great attention in recent years (Triroj et al., 2011; Huang and Mason, 2013; Wang et al., 2015; Lee et al., 2017; Baradoke et al., 2019; Gao et al., 2019; Kim et al., 2019; Yokus et al., 2020) and proved to possess the capacity to integrate with point-of-care systems. During implantation and long-term conditions, the performance to sample in real time is vital to the effective sensing. Utilizing low density aligned nano electrode arrays as robust transducer elements, Triroj et al. (2011) fabricated a microfluidic biosensor chip with improved sensitivity in a nanoliter volume testbed for the current response by two orders of magnitude compared to that obtained from a microelectrode. The nano electrodes arrays were functionalized with prostate specific antigen (PSA) to construct competitive immunoassay chip, and the detection limit is around 10 pg/mL (~270 fM), corresponding to ~30,000 copies of PSA. An improved sensitive functional system was developed by Wang et al. (2015) for wireless rapid analysis of saxitoxin and brevetoxin with portable cardiomyocyte-based potential biosensor. It was constructed by 8-channel recording micro electrode arrays, and can dynamically monitor the multisite electrical activity of cardiomyocyte network. Furthermore, a sensor based flexible microneedle electrode array, coupled with a multi-channel portable electrochemical analyzer, was developed by Gao et al. (2019) for the simultaneous detection of glucose, uric acid, and cholesterol levels in serum. Excellent sensing performance with a wide linear range, low detection limit and rapid response time was shown, therefore facilitating effective monitoring of blood metabolites at home. Nowadays, portable miniaturization micro/nano electrode arrays and wearable intelligent devices have showed the function of label-free, multi-parameter and real-time smart dynamic sensing, which play an important role in both the development of medical devices and biomedical research. In the future, smart sensing created from miniaturized micro/nano electrode array sensors would have broad prospects in biological applications, such as cellular behavior measurement, metabolism monitoring as well as new treatments development.

## Conclusions

This minireview mainly summarizes the recent advances in fabrication of micro/nano electrode array sensors and presents their emerging biological applications and their use in portable intelligent devices. In past decades, there have been remarkable progresses on the development of micro/nano electrode array sensors for biological applications, however, challenges still present. First, the miniaturized fabrication of micro/nano electrode arrays with more integrating multiplex (e.g., electro-optical response, wearable device) and the development of versatile sensors applicable to the actual conditions. Second, the influence of complex physiological environment *in vivo* on sensing sensitivity and selectivity remains an issue. Finally, sensing at small amounts of molecules and even exploring the relationship between molecular structure and function are expected eagerly. Finding out solutions to these challenges would be helpful for improving the stability and veracity of the detecting result in quality and quantity, and largely accelerating the wide application of micro/nano electrode array sensors in biological analysis. More importantly, it will greatly promote the understanding of physiological and pathological processes connected with matter in chemical movement, offering a unique contribution to the life related chemicals study and life science research.

## Author Contributions

All authors listed have made a substantial, direct and intellectual contribution to the work, and approved it for publication.

## Conflict of Interest

The authors declare that the research was conducted in the absence of any commercial or financial relationships that could be construed as a potential conflict of interest.
